# Glutathione has cell protective and anti-catabolic effects in articular cartilage without impairing the chondroanabolic phenotype

**DOI:** 10.1016/j.heliyon.2024.e40368

**Published:** 2024-11-13

**Authors:** Svenja Maurer, Michael Fuchs, Rolf E. Brenner, Jana Riegger

**Affiliations:** a Division for Biochemistry of Joint and Connective Tissue Diseases, Department of Orthopaedics, University of Ulm, Ulm, Germany; b Department of Orthopaedic Surgery, University of Ulm, Ulm, Germany

## Abstract

Joint injuries and consequent oxidative stress is a high-risk factor for developing post-traumatic osteoarthritis (OA). While antioxidative therapy using N-acetylcysteine (NAC) has cell- and chondroprotective effects following cartilage injury, it strongly impairs matrix synthesis. Consequently, direct application of Glutathione (GSH) was tested as an alternative therapeutic approach using an *ex vivo* cartilage trauma model and isolated chondrocytes, with comparison to NAC.

Porcine cartilage explants were traumatized using a drop tower with an impact energy of 0.47 J and afterwards treated with 0.5–2 mM GSH or 2 mM NAC for 4 days according to a standardized protocol. The effects of antioxidative treatment on the chondrogenic phenotype were tested in a 3D pellet culture for 28 days. Our results demonstrated that both antioxidants had cell protective effects after cartilage trauma. GSH was most effective at a concentration of 0.5 mM, as confirmed in experiments with isolated human chondrocytes exposed to H_2_O_2_. At this concentration, GSH did not impair cell proliferation or hyaline cartilage matrix synthesis, while NAC suppressed the chondrogenic phenotype in pellet culture. Both, NAC and GSH elevated the intracellular GSH concentration, indicating an efficient uptake of the antioxidants. Furthermore, both therapeutics inhibited the activity of the matrix degrading enzyme MMP-2.

Our results demonstrated cell- and chondroprotective effects by NAC and GSH therapy after cartilage trauma, with GSH demonstrating advantages in preserving the chondrogenic phenotype.

## 1 Introduction

The age-related disorder osteoarthritis (OA) is known as the most common joint disease worldwide [1,2]. Further risk factors include, inter alia, gender, genetic predisposition, ethnicity, obesity, diet or joint injuries [2]. Besides cartilage trauma, the latter comprises among others, ruptures of the cruciate ligaments, meniscal tears, patellar dislocations, and other bony lesions, which can promote post-traumatic OA (PTOA) [3,4]. This form of OA particularly affects young, physically active patients [1,3,4]. Joint injuries lead to necrotic and regulated cell death of chondrocytes and the release of damage-associated molecular patterns (DAMPs). This in turn elevates inflammation, enhances MMP expression, and leads to oxidative stress [4]. Pro-inflammatory cytokines, together with reactive oxygen and nitrogen species (ROS/RNS), play a crucial role in cell fate decision and phenotypic alterations in PTOA [5]. Consequently, a decline in chondroanabolic processes and an accumulation of dysfunctional, senescent chondrocytes can be observed, which contribute to ongoing cartilage degeneration. Taken together, oxidative stress is considered as a major driver of OA [4,5]. ROS/RNS can be produced by several organelles, such as the mitochondria, or enzymes like the NADPH oxidases (NOX) and NO synthase [5]. In fact, it has been reported that cartilage injury results in mitochondrial dysfunction and increased expression of ROS-producing enzymes [6]. The cellular antioxidative defense system for neutralizing ROS consists, among other enzymes, of catalases (CATs), superoxide dismutases (SODs), and glutathione (GSH) peroxidase. Further non-enzymatic antioxidants include GSH, vitamin E, and C. The balance of the intracellular redox homeostasis is of great importance, regarding the detrimental effects of mitochondrial dysfunction and subsequent excessive ROS production on cartilage integrity [5,7].

To reduce the harmful effects of oxidative stress after joint and cartilage injury, several antioxidants have already been tested *in vitro* and *in vivo* [5,8]. In this context, the synthetically produced N-acetylcysteine (NAC) has been intensively studied [[9], [10], [11], [12], [13]] and was found to possess cell- and chondroprotective effects in rabbit [12], rat [9,10], and human PTOA models [11,13]. NAC directly scavenges ROS through its thiol group, breaks protein disulfide bonds, and acts as an indirect antioxidant as a precursor for cysteine, which is necessary for GSH *de novo* synthesis [14,15], a tripeptide consisting of cysteine, glutamate, and glycine [16]. The intracellular concentration of GSH ranges from 0.5 to 10 mM. The tripeptide is quite stable against hydrolysis due to a specific peptide bond [17]. Like NAC, GSH can directly scavenge ROS/RNS, or can be used as a precursor for antioxidative enzymes. GSH peroxidases, for example, use GSH to reduce H_2_0_2_, which is primarily generated in mitochondria through the SOD2-driven elimination of superoxide. Consequently, GSH is reduced to glutathione disulfide (GSSG) which can be recovered by the enzyme GSH reductase [5].

Despite the beneficial effects of NAC after cartilage trauma, our previous studies demonstrated impairing effects of NAC on chondroanabolism [11,13,18]. NAC-mediated suppression of collagen type 2 biosynthesis in cartilage and during chondrogenic differentiation of cartilage-derived progenitor cells was even maintained in a multimodal therapeutic approach, in which the antioxidant was combined with potential anabolic growth factors [13,18].

In our current study, we aim to evaluate whether the antioxidant GSH could be applied for damage reduction without impairing chondroanabolism after cartilage injury. For this purpose, we used a drop tower to traumatize porcine cartilage explants [11,19], and observed cell protective effects for NAC and GSH treatment. While NAC therapy resulted in a strong suppression of the chondrogenic phenotype of porcine articular chondrocytes (pAC), GSH treatment inhibited hyaline matrix production to a much lesser extent. Furthermore, GSH and NAC displayed anti-catabolic effects through direct inhibition of the MMP-2 enzymatic activity. Measurement of the intracellular total GSH amount confirmed the uptake of both, GSH and NAC. Our results indicate that GSH supplementation could represent a potential add-on therapy for the prevention of PTOA.

## 2 Material and methods

### 2.1 Isolation and cultivation of chondrocytes and cartilage explants

Cartilage explants (Ø 6 mm) were isolated from femoral condyles of porcine knee joints (local butcher, 4–8 months old) and cultivated in serum-free medium [DMEM 4.5 g/L glucose (Gibco Life Technologies), 1 % Na-pyruvate (Merck Biochrom), 1 % NEAAs (Bio&Sell), 2 mM L-glutamine (PAN-Biotech), 1 % penicillin/streptomycin (PAN-Biotech), 10 μg/mL 2-phospho-L-ascorbic acid trisodium salt (Sigma-Aldrich), 1 % 100x ITS (Gibco, Life Technologies)]. Human cartilage tissue was obtained from patients after written permission during total knee arthroplasty surgery (ethical approval number: 353/18 given by the Ethics Commitee of the University Ulm). Isolation of chondrocytes was performed using 0.2 % protease (from streptomyces griseus, Sigma, P6911) for 45 min at 37 °C, and 0.025 % collagenase (from clostridium histolyticum, Sigma, C5138) at 37 °C overnight. Afterwards, the chondrocytes were filtered through a cell strainer and seeded in chondrocyte full medium [50:50 Ham's F12 (PAN-Biotech) and DMEM, 10 % heat inactivated fetal bovine serum (FBS) (PAN-Biotech), 0.5 % penicillin/streptomycin, 2 mM L-glutamine, 10 μg/mL 2-phospho-L-ascorbic acid trisodium salt]. In case of porcine articular chondrocytes (pAC), DMEM containing 4.5 g/L glucose was used, while DMEM for hAC contained 1 g/L glucose. During the experiments, isolated cells were cultivated in serum-reduced medium (50:50 serum-free and chondrocyte full medium) [11,20].

### 2.2 Traumatization of cartilage explants

After a recovery time of at least 2 days after preparation of the porcine cartilage explants, the tissue was traumatized using our drop tower approach (impact energy: 0.47 J) [11,19] and subsequently treated with 2 mM NAC (Sigma Aldrich, A9165) or 0.5 – 2 mM GSH (Sigma Aldrich, G4251) for 4 d. Antioxidants were refreshed with medium change 48 h after cartilage trauma. Analysis was performed at day 4, and 3 days after deprivation of the antioxidants (at day 7).

### 2.3 Cell viability analysis – live/dead staining

4 and 7 d after trauma, cell viability of cartilage explants was determined using the LIVE/DEAD™ Viability/Cytotoxicity Kit (Invitrogen, L3224) as previously described [11]. In brief, thin cartilage sections of 0.5 mm were excised using a double-bladed scalpel. Cartilage sections were stained for 30 min at RT with 1 μM calcein AM (live cells appear in green) and 2 μm ethidiumhomodimer-1 (dead cells appear in red). Afterwards, samples were transferred into PBS and images were taken by means of a z-stack with the Zeiss Microscope Axioskop 2 mot plus (Software AxioVision). Live and dead cells were counted manually using the software Fiji/ImageJ 2.14.0/1.54f.

### 2.4 Staining apoptotic cells – TUNEL assay

For analysis of apoptotic cells in cartilage tissue, the DeadEnd™ Fluorometric TUNEL System (Promega, GE3250) was used according to the manufacturer's instruction. Apoptotic cells are visualized by green fluorescence and nuclear DAPI staining was used to detect all cells.

### 2.5 Cytotoxicity analysis – alamarBlue assay

Isolated hAC were exposed to 100 μM H_2_0_2_ for 48 h with or w/o GSH preincubation (0.5–4 mM, overnight) or stimulated with GSH (0.5–4 mM) and H_2_0_2_ simultaneously (48 h). Untreated cells served as control group. To estimate the cell viability, an alamarBlue assay (Bio-Rad, BUF012A) was performed, in which viable cells metabolize the non-fluorescent dye resazurin (alamarBlue) into the fluorescent dye resorufin. After washing with PBS, cells were incubated with 5 % alamarBlue staining solution in an incubator at 37 °C for 3 h. Afterwards, the fluorescence intensity was assessed (excitation: 550 nm, emission: 590 nm) by means of a Tecan Microplate Reader infinite M200 pro [20].

### 2.6 GSH/GSSG assay

To investigate the uptake of the antioxidants, hAC were treated with GSH (0.5–2 mM) or NAC (2 mM) for 24 h. Cells were detached by trypsin, centrifuged, resuspended in PBS, and counted. According to the assay protocol (Elabscience, E-BC-K097-M.96, Total Glutathione (T-GSH)/Oxidized Glutathione (GSSG) Colorimetric Assay Kit), cells were centrifuged at 1000 g (10 min) and cell sediment was resuspended with the protein precipitator at a concentration of 10^6^ cells per 400 μl. Subsequently, samples were sonicated, centrifuged at 10,000 g (10 min), and supernatants were preserved on ice. The preparation of the standard and working solutions, as well as the measurement of T-GSH and GSSG were performed according to the manufacturer's instruction.

### 2.7 Cell proliferation and migration analysis – gap assay

To determine cell proliferation and undirected migration, hAC were seeded into the wells of a culture insert (ibidi 80209). After adhesion of the cells, the insert was removed and hAC were stimulated with 0.5–2 mM GSH. Images of the gap were taken directly after stimulation (t0), after 24 h and 48 h using a light microscope (Zeiss, Axiovert 35). For analysis, cells in the gap were counted manually (24 h) or the overgrown area was determined (48 h) using the software Fiji/ImageJ 2.14.0/1.54f.

### 2.8 Cell proliferation analysis – Ki67 immunofluorescence staining

hAC were seeded on Nunc Lab-Tek chamber slides and stimulated with 0.5–2 mM GSH or 2 mM NAC for 48 h. The proliferation was studied by means of a Ki67 immunofluorescence (IF) staining. In short, cells were fixed with 4 % formalin, permeabilized with 0.1 % Triton-X-100, blocked for 1 h at 37 °C (Dako Blocking Buffer) and stained with the primary antibody at 4 °C overnight (Ki67-AB, ab16667, rabbit anti-human/mouse/rat IgG, dilution 1:250). Secondary antibody (Alexa Fluor® 488, ab150077, goat anti-rabbit IgG, dilution 1:200) was applied for 30 min at room temperature (RT) and nuclei staining was performed using DAPI (15 min, RT). Subsequently, fluorescence mounting medium (FluorSave™ Reagent, Merck) was applied and images were taken using the fluorescence microscope (Zeiss Microscope Axioskop 2 mot plus).

### 2.9 Gelatin zymography

To investigate direct inhibitory effects of the antioxidants on the proteolytic MMP-2 activity, a gelatin zymography was performed. Supernatants of cartilage explants containing MMP-2 were pre-incubated with 10 mM NAC or 10 mM GSH. Same volumes of the supernatants without antioxidants served as control group to demonstrate the proteolytic activity of the gelatinase. The samples were mixed with sample buffer (Bio-Rad) and applied onto a 10 % polyacrylamid gel containing 2 mg/mL gelatin (Merck). The electrophoresis was undertaken at 4 °C. Afterwards, the gel was incubated in renaturation buffer (Bio-Rad) for 30 min at RT and subsequently incubated overnight in development buffer according to the Bio-Rad recipe [50 mM Tris-HCl (Roth), 200 mM NaCl (AppliChem), 5 mM CaCl_2_ (Merck), 0.02 % Brij-35 (Merck)] at 37 °C. The gel was stained using Coomassie R250 Brilliant Blue (Fluka Chemika) staining solution and pro MMP-2 and active MMP-2 bands were revealed by incubation in a de-staining buffer [6,11].

### 2.10 3D pellet culture

To study the maintenance of the chondrogenic phenotype, 3.5•10^5^ pAC in passage 1–2 were centrifuged and cultivated in a 3D pellet culture for 28 days in chondrogenic differentiation medium (CDM) [DMEM 4.5 g/L glucose, 1 % penicillin/streptomycin, 1 % ITS, 2 mM L-Glutamine, 0.1 μM Dexamethasone (Sigma-Aldrich), 0.2 mM 2-phospho-L-ascorbic acid trisodium salt, 40 μg/mL L-proline, 1 mM Na-pyruvate, 10 ng/mL of rhTGF-β3 (PeproTech, 100-36E) and 10 ng/mL rhBMP-6 (PeproTech, 120-06)]. The cell pellets were treated with 2 mM NAC (for 7 or 28 days), or 0.5 – 1 mM GSH (for 7 or 28 days). Medium, containing supplements and antioxidants, was changed thrice a week. Afterwards, pellets were embedded in paraffin and a Safranin O (SafO) staining was performed as described below. Assessment of the chondrogenic phenotype, regarding hyaline matrix production and cell morphology, was performed by using the scoring system of Ruths et al., as previously reported [18,21].

### 2.11 Histology – Safranin O staining

For histological analysis of cartilage (7 d after trauma) and cell pellets, the samples were fixed with formalin 4 %, dehydrated, and embedded in paraffine. To perform SafO staining, 4 μm thick sections were rehydrated, and subsequently, the nuclei were stained with Weigert's iron hematoxylin (6 min). Proteoglycans were stained using 0.1 % SafO staining solution (6 min) (Chroma-Gesellschaft) and 0.02 % fast-green (3 min) (Sigma Chemical Company) was used as a counterstaining. Images were taken using a Zeiss Microscope Axioskop 2 mot plus [18].

### 2.12 Statistics

For statistical analysis the GraphPad Prism Software 10 was used. Data sets n *≥* 5 were tested for outliers (Grubb's outlier test), which were not included in the statistical analysis. One-way ANOVA was used to compare 3 or more groups. The significance level was set to *α* = 0.05. Detailed statistical information is provided in the captions.

## 3 Results

### 3.1 NAC and GSH exert cell protective effects after *ex vivo* cartilage trauma

**Fig. 1:**
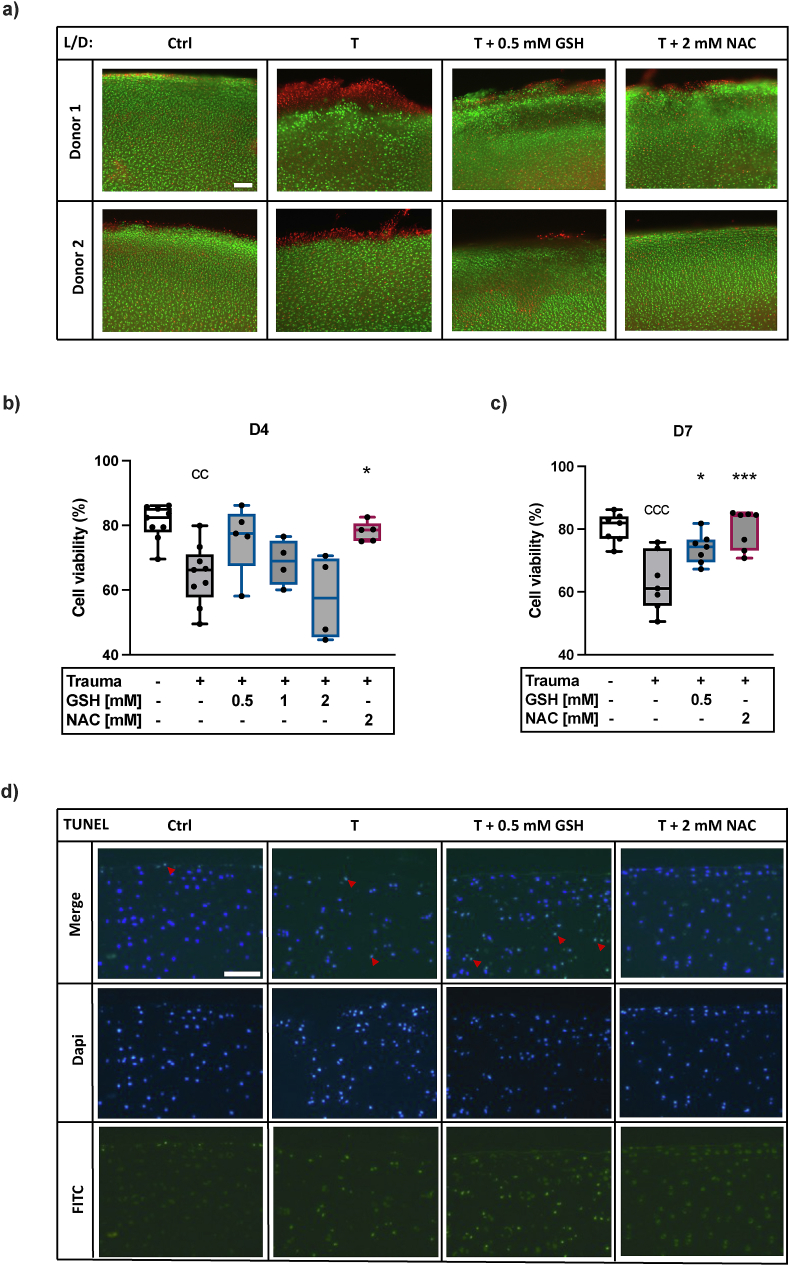
**Cell protective effects of NAC and GSH after cartilage trauma**. a) Exemplary live/dead staining of unimpacted and traumatized cartilage w/or w/o NAC (2 mM) or GSH (0.5 mM) treatment, scale bar = 200 μm. b) Corresponding cell viability (%) of unimpacted control cartilage as well as traumatized cartilage w/or w/o 2 mM NAC or 0.5–2 mM GSH therapy 4 d after trauma (n ≥ 4) c) and at d7 (n = 7) (3 days after deprivation of the antioxidants). d) Exemplary TUNEL staining (d7) (scale bar = 100 μm), red arrowheads exemplarily indicate TUNEL-positive cells. Statistical analysis: b) + c) One-way ANOVA with Dunnett's multiple comparisons test: ∗p < 0.05, ∗∗∗p < 0.001 (vs. Trauma); ^cc^p<0.01, ^ccc^p<0.001 (vs. Ctrl).

Traumatization of the explants with 0.47 J resulted in a significant reduction of the cell viability (−16 %) at d4. Treatment with low concentration of GSH (0.5 mM) increased the cell viability after trauma (+11 %), while high concentrations of GSH (≥1 mM) exhibited no cell protective effects. NAC (2 mM) provided comparable cell protection (+13 %) (Fig. 1b). Three days after deprivation of the antioxidants (d7), the live/dead staining demonstrated similar cell protective effects after treatment with 0.5 mM GSH (+11 %) and 2 mM NAC (+17 %) (Fig. 1a + c). Overall, NAC therapy achieved better results regarding cell protection, which was confirmed by means of a TUNEL assay (d7), detecting apoptotic cells. The control displayed only a few apoptotic cells, while trauma induced apoptosis of chondrocytes. Therapy with 0.5 mM GSH could not prevent cells undergoing apoptosis, while NAC distinctly reduced the number of apoptotic chondrocytes after trauma (Fig. 1d).

### 3.2 NAC and GSH directly inhibit the activity of MMP-2

**Fig. 2:**
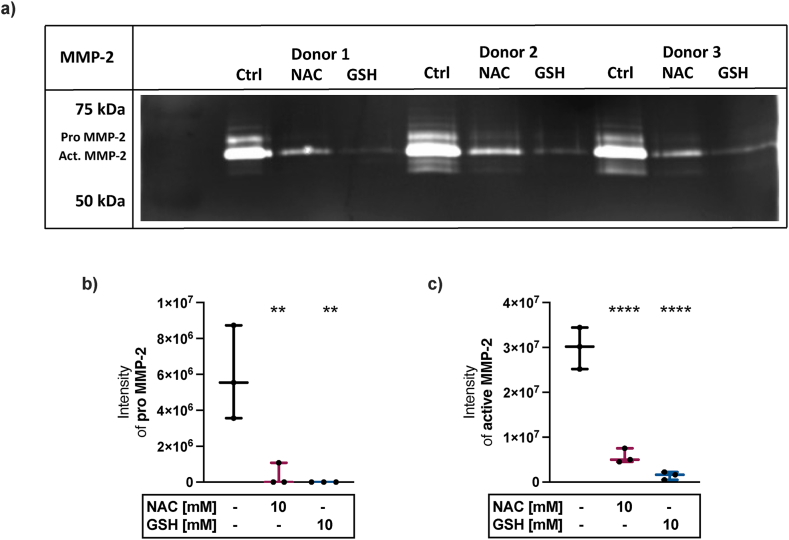
**Direct inhibition of MMP-2 activity by NAC and GSH.** a) Gelatin zymography gel with supernatants of 3 experiments and pre-incubation with 10 mM NAC, 10 mM GSH or w/o antioxidants. Gamma adjustment was applied to visualize faint bands (Image Lab 6.1; BioRad). An uncropped and unadjusted image of the gel is provided in **S1**. b) Band intensities of pro MMP-2 and c) active MMP-2. Statistical analysis: One-way ANOVA with Dunnett's multiple comparisons test: ∗∗p < 0.01, ∗∗∗∗p < 0.0001 (vs. untreated Ctrl).

The gelatinase A (MMP-2) contributes to cartilage degeneration through cleavage of extracellular matrix components [22]. Therefore, the enzymes’ activity was investigated by means of a gelatin zymography. Direct addition of NAC or GSH to the supernatants of cartilage explants prior to the zymography resulted in a significant reduction of the proteolytic activity of both pro MMP-2 and active MMP-2. Overall, GSH displayed stronger inhibitory effects than NAC (Fig. 2a–c).

### 3.3 NAC and GSH have no effect on the histomorphologic characteristics after *ex vivo* cartilage trauma

**Fig. 3:**
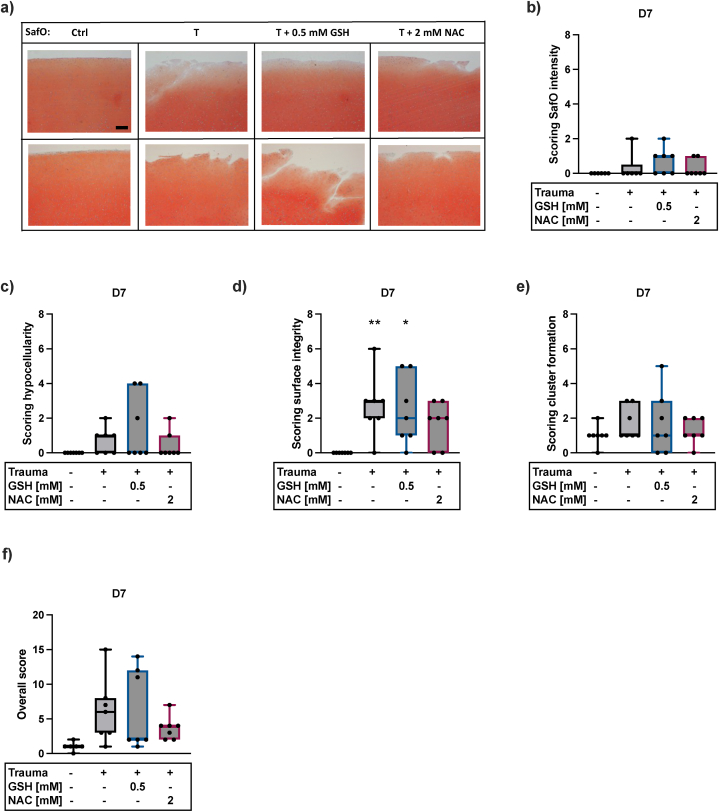
**Effects of trauma and antioxidative therapy on cartilage histomorphology**. a) Exemplary images of SafO staining of 2 donors. Scoring of b) SafO intensity (n ≥ 6), c) hypocellularity (n = 7), d) surface integrity (n = 7), e) cluster formation (n = 7), and f) overall score (n ≥ 6). Statistical analysis: One-way ANOVA with Dunnett's multiple comparisons test: ∗p < 0.05, ∗∗p < 0.01 (vs. w/o trauma).

Based on a modified Mankin score, the histomorphology of cartilage tissue was analyzed after SafO staining. The four categories – surface integrity, hypocellularity, SafO staining intensity, and cluster formation – were scored from 0 to 6, while a higher score represents a reduction in cartilage quality. Cartilage trauma increased hypocellularity and chondrocyte cluster formation by trend and resulted in a significant destruction of the cartilage surface integrity (Fig. 3c–e). However, SafO staining intensities were not affected by trauma (Fig. 3a + b). Neither NAC, nor GSH could significantly prevent trauma-related hypocellularity, cluster formation, or destruction of the cartilage surface integrity (Fig. 3c–e). Nevertheless, therapy with NAC reduced the overall score by trend (Fig. 3f).

### 3.4 NAC has stronger impairing effects on the chondrogenic phenotype than GSH

**Fig. 4:**
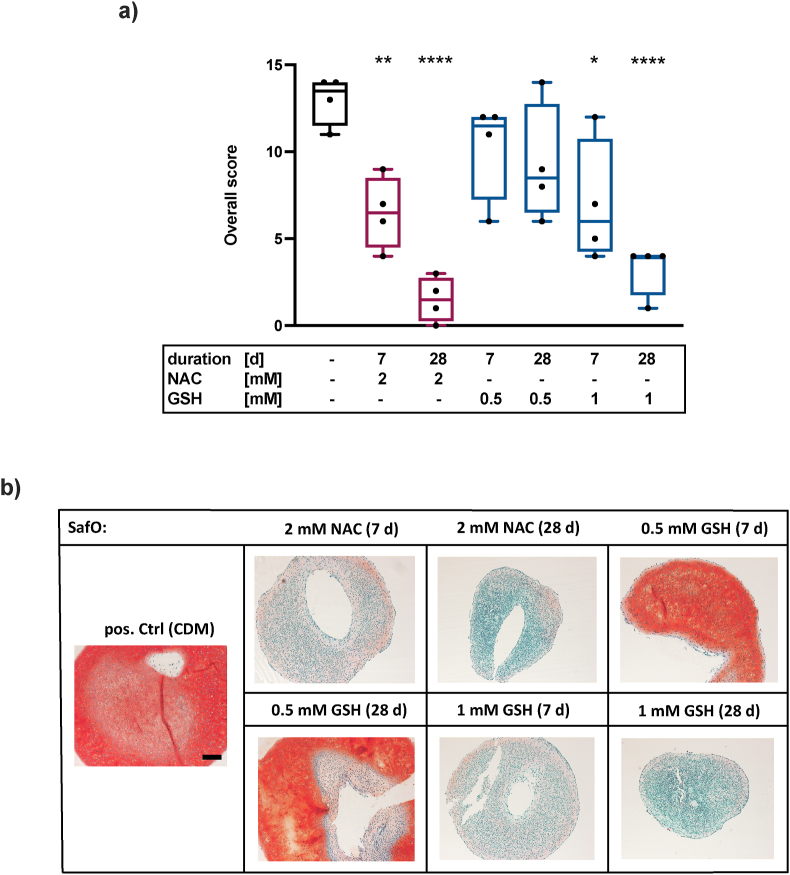
**Effects of NAC and GSH on the chondrogenic phenotype of pAC.** a) Overall score of the chondrogenic phenotype with GSH (0.5 mM–1 mM), NAC (2 mM) or w/o antioxidants (n = 4). b) Exemplary images SafO staining, scale bar = 200 μm. Statistical analysis: One-way ANOVA with Dunnett's multiple comparisons test: ∗p < 0.05, ∗∗p < 0.01, ∗∗∗∗p < 0.0001 (vs. pos. Ctrl (CDM)).

In previous studies, we described strong impairing effects of NAC on the chondrogenesis of human chondrogenic stem/progenitor cells (CSPCs) [18] and gene expression of COL2A1 in human cartilage tissue [11,13]. Therefore, we used pAC and investigated if GSH treatment exerts similar detrimental effects on the chondrogenic phenotype as found for NAC. Addition of NAC for 7 d already reduced the matrix production to a great extent. Continuous treatment for 28 d distinctively suppressed the chondrogenic phenotype, which was reflected in a significant decrease of the overall score. On the contrary, 0.5 mM GSH for 7 d and even for 28 d had less disturbing influence on matrix production. 1 mM GSH in short- or long-term application displayed similar impairing effects as observed for NAC (Fig. 4a–b).

### 3.5 Uptake of extracellular GSH by hAC protects cells from H_2_0_2_-induced cytotoxicity

**Fig. 5:**
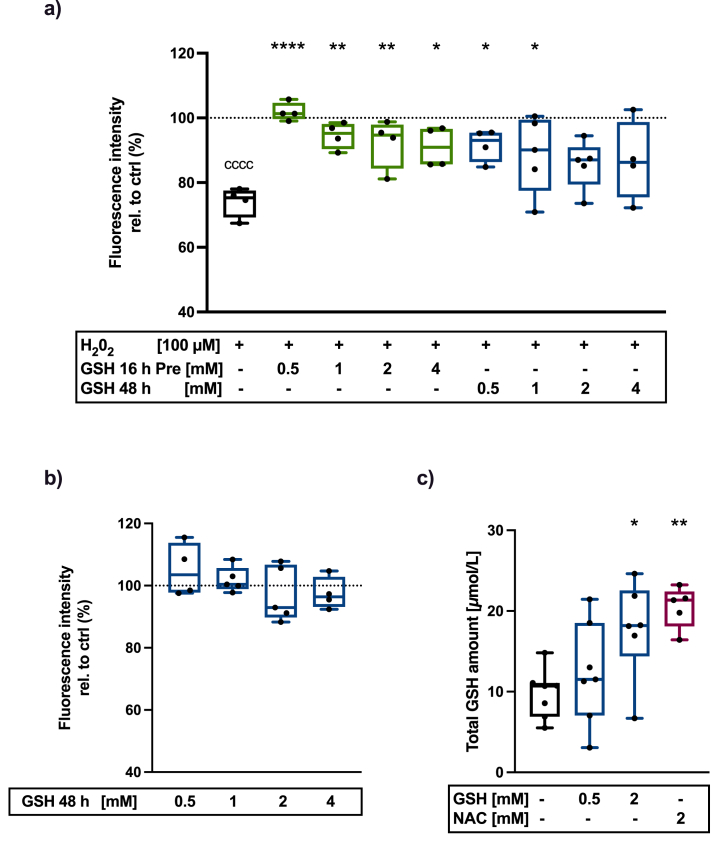
**Cell protective effects of GSH are mediated through its cellular uptake.** a) FIs of an alamarBlue assay 48 h after H_2_0_2_ exposition (100 μM) w/or w/o pre-incubation (16 h) or simultaneous addition of GSH (0.5–4 mM) (n ≥ 4). b) FIs (alamarBlue) of hAC stimulated with GSH (0.5–4 mM) (n ≥ 4). c) Total GSH amount after GSH (0.5 – 2 mM) or NAC (2 mM) treatment for 24 h (n ≥ 5). Statistical analysis: a) One-way ANOVA with Dunnett's multiple comparisons test: ∗p < 0.05, ∗∗p < 0.01, ∗∗∗∗p < 0.0001 (vs. H_2_0_2_ treatment), c) one-way ANOVA with Dunnett's multiple comparisons test: ∗p < 0.05, ∗∗p < 0.01, (vs. Ctrl).

In order to clarify if cell protective effects of GSH result from neutralization of intracellular or extracellular ROS, we exposed hAC to 100 μM H_2_0_2_ in different experimental settings. Addition of H_2_0_2_ resulted in a significant reduction of the metabolic activity (−26 %), measured by means of an alamarBlue assay. Pre-incubation with GSH (0.5–4 mM) for 16 h and subsequent addition of H_2_0_2_ w/o GSH treatment prevented cytotoxic effects on hAC. Similar results were observed for simultaneous administration of GSH (0.5–4 mM) and H_2_0_2._ Overall, the most efficient cell protection by GSH was found at a concentration of 0.5 mM (Fig. 5a). GSH alone had no influence on the FI, demonstrating no cytotoxic effects up to a concentration of 4 mM (Fig. 5b). As pre-incubation protected hAC from H_2_0_2_, we assumed that GSH was taken up by the cells. This hypothesis was confirmed by means of a T-GSH/GSSG-Assay. The intracellular T-GSH amount was significantly increased after GSH treatment (2 mM) compared to the untreated control group. Similarly, NAC therapy resulted in enhanced T-GSH levels, confirming its uptake and use for the *de novo* synthesis of GSH (Fig. 5c). The levels of cytotoxic GSSG were below the limit of detection, thus the measured T-GSH concentration represents the reduced GSH concentration.

### 3.6 GSH inhibits the cell proliferation and migration of hAC at high concentrations

**Fig. 6:**
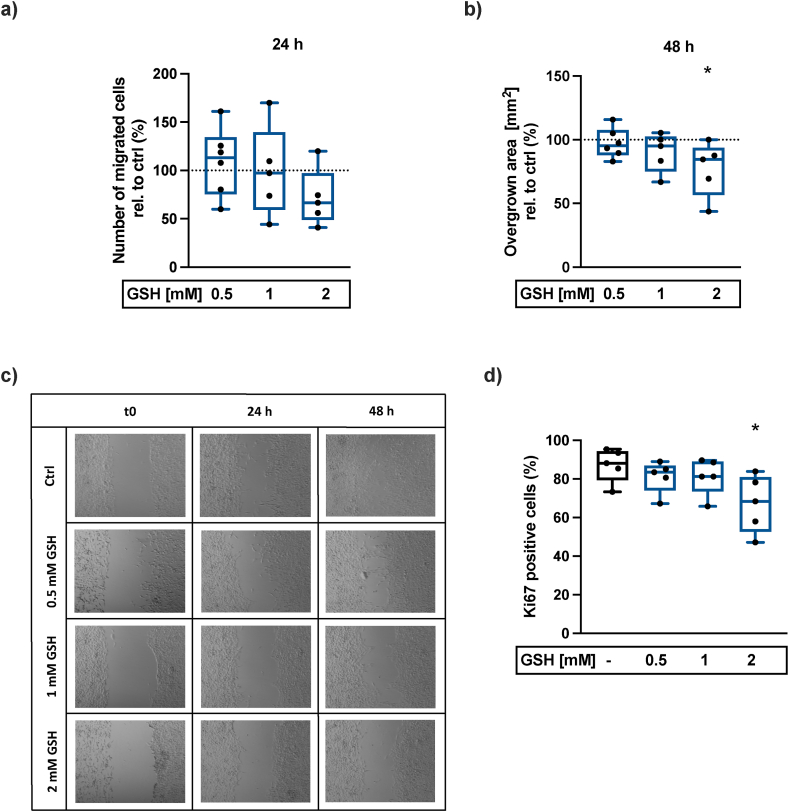
**GSH inhibits undirected cell migration and proliferation in a concentration-dependent manner.** a) Relative number of migrated chondrocytes 24 h after GSH stimulation (0.5–2 mM) (n ≥ 5) and b) the corresponding determination of the overgrown area after 48 h (n ≥ 5). c) Exemplary images of the gap after 0 h, 24 h and 48 h w/or w/o GSH stimulation (0.5–2 mM). d) Ki67 IF staining of hAC w/or w/o GSH for 48 h (0.5–2 mM) (n = 5). Statistical analysis: One-way ANOVA with Dunnett's multiple comparisons test: ∗p < 0.05 (vs. untreated Ctrl).

GSH therapy displayed a concentration-dependent inhibitory effect on undirected cell migration, which was determined by a gap assay after 24 h and 48 h (Fig. 6a–c). Additionally, Ki67 IF staining displayed anti-mitotic effects of high concentrations (2 mM) of GSH (Fig. 6d).

## 4 Discussion

Oxidative stress is a main driver of OA and augmented oxidative stress levels have accordingly been detected in patients suffering from this joint disease [7,[23], [24], [25]]. Low ROS levels in chondrocytes are necessary to maintain the highly regulated cartilage homeostasis. Excessive levels on the contrary, contribute to mitochondrial dysfunction, chondrocyte senescence, cellular apoptosis, cartilage degeneration, dysfunction of subchondral bone, and synovitis [7]. Therefore, antioxidative therapy could offer a potential therapeutic treatment to reduce the risk of developing a PTOA upon joint injury or to delay OA progression in general, as previously demonstrated for NAC [[9], [10], [11], [12]].

In this study, we tested the therapeutic effects of GSH for the first time in cartilage explants after trauma and compared the results with the well-described antioxidant NAC. The present results clearly demonstrated cell protective effects for both antioxidants. Moreover, the antioxidants revealed anti-catabolic effects through direct inhibition of the proteolytic activity of MMP-2. While NAC strongly impaired the chondrogenic phenotype of pAC in 3D pellet culture, GSH had only minor suppressive effects at its ideal concentration. Moreover, GSH uptake by hAC protected the cells against H_2_O_2_-related oxidative stress.

The cell- and chondro-protective effects of NAC observed in this study are in line with previous efforts from our group, in which NAC was tested in a human cartilage trauma model [11]. Several *in vivo* studies have already been performed using NAC in rat [9,10], rabbit [12] and porcine [26] PTOA models, while GSH therapy has not yet been tested in any *in vivo* or *ex vivo* PTOA model.

This is the first study highlighting that GSH exerts similar cell protective effects as compared to NAC. However, it should be noted that GSH-mediated cell protection was only found for low concentrations (0.5 mM), while increasing concentrations were less effective. Nevertheless, studies in other biologic context reported about beneficial effects of GSH at a concentration of 1–10 mM [[27], [28], [29], [30]]. Moreover, cell protective effects of NAC and GSH remained preserved for at least 3 days after deprivation of the antioxidants. Cell protection of NAC and GSH could be explained by the support of the intracellular defense system. High intracellular GSH levels are thought to promote the apoptosis resistance of cells. Depletion of GSH, on the other hand, induces apoptosis via extrinsic and intrinsic signaling pathways [16,31]. Intracellular GSH concentrations affect the expression of cell death-regulating signaling molecules and caspases. A decrease in intracellular GSH concentration can be found in cells undergoing apoptosis, while an excessive decrease in GSH shifts the mode of cell death towards necrosis [32]. Accordingly, Cheng et al. reported protective effects of a GSH-loaded chitosan-gelatin-based hydrogel against oxidative stress in chondrocyte-like cells affected by mitochondrial dysfunction [33]. Promotion of the intracellular GSH antioxidative defense system in OA might be beneficial considering the findings of Carlo et al. that chondrocytes of elderly patients (≥50 years) exhibit a higher GSSG/GSH ratio compared to younger individuals (<50 years) [34]. Our findings indicate that uptake of exogenous GSH by hAC is possible. The uptake of GSH in chondrocytes might occur via amino acid transporters. This could be achieved by the enzymatic breakdown of GSH into cysteine, glycine, and γ-glutamyl amino acid on the cell surface, and subsequent uptake of the separate amino acids via transporters. After internalization, cells can use the precursor amino acids for *de novo* synthesis of GSH [16]. Nevertheless, there are studies of GSH transporters in lens epithelial cells for uptake of circulating GSH in its original molecular state [35]. Additionally, Lash et al. reported about GSH uptake across the basolateral plasma membrane via organic anion transporter 3 in renal proximal tubule cells [36]. Therefore, the exact mechanism of extracellular GSH uptake by hAC requires further investigation.

In line with our findings that GSH displays no suppression on hyaline matrix production in chondrocytes, Yang et al. reported that GSH exerted no detrimental effects on the glycosaminoglycan synthesis of human fibroblast-like synoviocytes [37]. As low concentrations of GSH are needed to protect cells from cell death, inhibition of the anabolism could be circumvented in an optimal concentration range, thereby maintaining the articular chondrocyte phenotype. Thus, GSH application may offer a significant advantage over NAC.

Moreover, we demonstrated that GSH and NAC exhibited anti-catabolic effects by direct inhibition of MMP-2, which is in agreement with the findings of Bogani et al. [38]. In addition, Pei et al. identified NAC and GSH as MMP-9 inhibitors. The inhibitory mechanism of the nonprotein thiols was explained through interaction with the Zn^2+^ within the catalytic site of the active center [39]. Furthermore, Sunitha et al. reported about analogous inhibitory effects on hyaluronidases by GSH and NAC [40].

Low GSH levels have been reported in other age-related disorders, in which oxidative stress is considered as an underlying pathomechanism, comprising diabetes mellitus, neurodegenerative-, and cardiovascular diseases. Therefore, maintaining optimal GSH levels could represent a potential strategy to prevent or treat these diseases [41,42]. The increase of GSH levels can be achieved by several methods: for instance, via application of liposomal GSH, precursors of GSH (NAC, γ-glutamylcysteine), GSH esters, or through Nuclear Factor Erythroid 2-Related Factor 2 (Nrf2) activating drugs, which induce the intracellular GSH synthetase (GSHS) and γ-glutamylcysteine synthetase (γ-GCS) [41]. However, the cellular uptake of the antioxidant NAC as a negatively charged molecule is slow and followed by the deacetylation to cysteine [43]. Moreover, oral application of GSH or NAC results in a poor bioavailability [41], thus a more sufficient approach to increase intracellular GSH is of great importance. In case of OA, intraarticular injection of GSH could be a conceivable method to prevent or attenuate disease progression after joint injury, as previously described for NAC *in vivo* [12]. Furthermore, future studies might focus on the application of GSH in combination with orthobiologic therapies, such as platelet rich plasma. To the best of our knowledge, exogeneous GSH administration has not been thoroughly investigated in the field of OA research. However, in other research fields, GSH treatment displayed beneficial effects, such as protection of gastric [28] and small-intestine epithelial cells under oxidative stress [29], promotion of *in vitro* oocyte maturation [27], and protection of proximal tubule epithelial cells during anoxia [30].

Our study is limited by the fact that porcine cartilage tissue was used which displays different regeneration capacities compared to human tissue. One remarkable difference is for instance the higher cell density in porcine tissue. In our study, experiments using isolated hAC were included to confirm that GSH also exerts beneficial effects on human OA cells. Moreover, our *ex vivo* cartilage trauma model does not consider the crosstalk with cells of the synovial membrane or subchondral bone, although OA is a disease of the entire joint.

Overall, we could demonstrate that GSH can be used as a cell protective drug after cartilage trauma like NAC. Both antioxidants revealed anti-catabolic effects and thus might prevent posttraumatic cartilage degeneration. Additionally, cell protective concentrations of GSH had no detrimental effect on the chondrogenic phenotype as observed for NAC. Based on our findings, GSH could be used as a potential novel antioxidative treatment in the context of posttraumatic harm reduction and subsequent prevention of OA development.

## CRediT authorship contribution statement

**Svenja Maurer:** Writing – original draft, Visualization, Validation, Investigation, Formal analysis, Data curation, Conceptualization. **Michael Fuchs:** Writing – review & editing, Investigation. **Rolf E. Brenner:** Writing – review & editing, Project administration, Funding acquisition, Data curation. **Jana Riegger:** Writing – original draft, Validation, Supervision, Project administration, Funding acquisition, Data curation, Conceptualization.

## Data and code availability statement

Data included in article/supplementary material is referenced in the article.

## Declaration of competing interest

The authors declare that they have no known competing financial interests or personal relationships that could have appeared to influence the work reported in this paper.
