# Supplementary figures and images for: Glutathione has cell protective and anti-catabolic effects in articular cartilage without impairing the chondroanabolic phenotype
Source: Heliyon. 2024 Nov 13;10(22):e40368. doi: 10.1016/j.heliyon.2024.e40368 (PMC11609657; doi:10.1016/j.heliyon.2024.e40368)

**Figure S1:** Original and uncropped zymography gel (MMP-2).

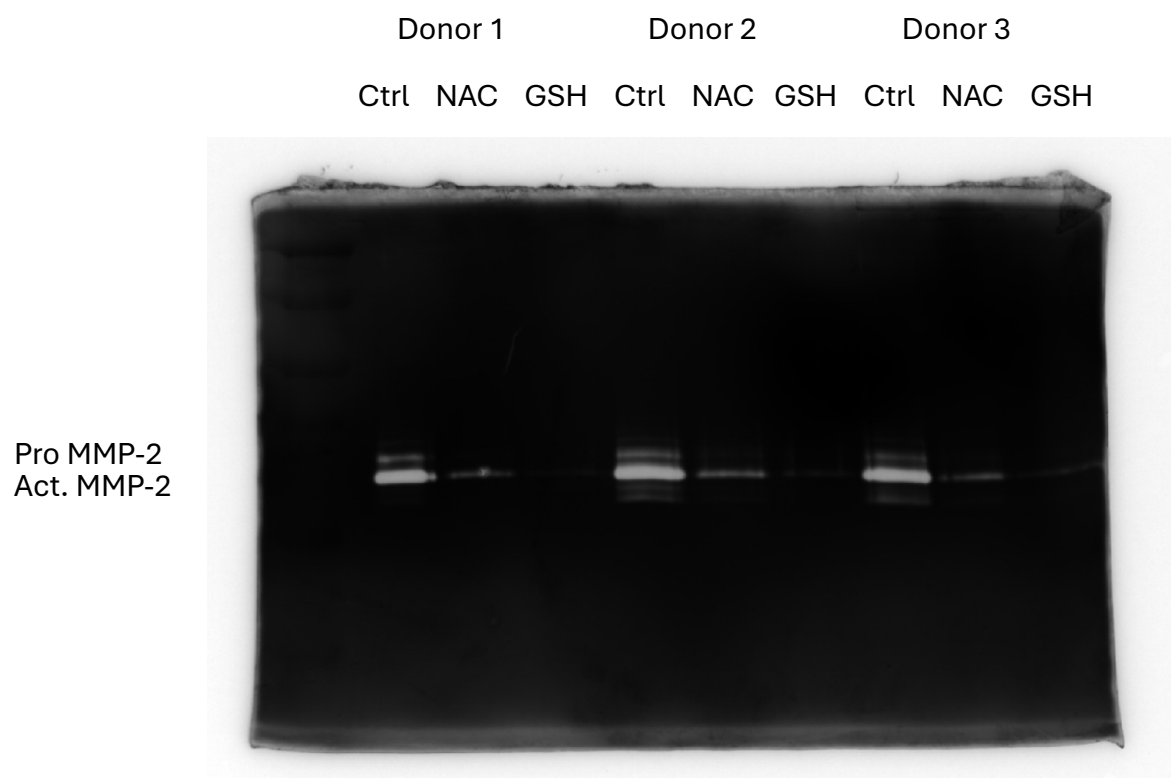

Supplement: Multimedia component 1 [file mmc1.pdf]
